# Retraction: MiR-25-3p targets PTEN to regulate the migration, invasion, and apoptosis of esophageal cancer cells via the PI3K/AKT pathway

**DOI:** 10.1042/BSR-2020-1901_RET

**Published:** 2024-01-31

**Keywords:** apoptosis, esophageal cancer, invasion, migration, miR-25-3p

This article is being retracted from *Bioscience Reports* at the request of the Editor-in-Chief and the Editorial Board following receipt of a notification from a reader, alerting the Editorial Board to an image in Figure 3C (Blank 48 hour panel) which is duplicated in Figure 11A from Zhao et al. 2021 (doi: 10.1186/s12935-020-01698-7).

The authors contacted the Editorial Office requesting to withdraw the article, but did not refer to the duplication, stating “further exploration and verification are required”. The authors were contacted regarding the concerns raised and asked to agree to the retraction, but did not respond.

